# Fourteen-year Clinical Performance of a HEMA-free One-step Self-etch Adhesive in Non-carious Cervical Lesions

**DOI:** 10.3290/j.jad.b4208859

**Published:** 2023-07-12

**Authors:** Marleen Peumans, Ellen Van de Maele, Jan de Munck, Kirsten van Landuyt, Bart Van Meerbeek

**Affiliations:** a Full Professor, KU Leuven (University of Leuven), Department of Oral Health Sciences, BIOMAT & UZ Leuven (University Hospitals Leuven), Dentistry, Leuven, Belgium. Wrote the manuscript, evaluated the restorations at the recalls.; b Master-after-Master in Restorative Dentistry (University of Leuven), Department of Oral Health Sciences, BIOMAT & UZ Leuven (University Hospitals Leuven), Dentistry, Leuven, Belgium. Performed the 14-year recall in partial fulfillment of requirements for the degree of Master-after-Master in Restorative Dentistry.; c Post-doctoral Research Fellow; KU Leuven (University of Leuven), Department of Oral Health Sciences, BIOMAT & UZ Leuven (University Hospitals Leuven), Dentistry, Leuven, Belgium. Performed the statistical analysis.; d Professor, KU Leuven (University of Leuven), Department of Oral Health Sciences, BIOMAT & UZ Leuven (University Hospitals Leuven), Dentistry, Leuven, Belgium. Idea, hypothesis, experimental design, follow-up of 1, 3 and 5 year recalls.; e Full Professor, KU Leuven (University of Leuven), Department of Oral Health Sciences, BIOMAT & UZ Leuven (University Hospitals Leuven), Dentistry, Leuven, Belgium. Proofread the manuscript, evaluated the restorations at the recalls.

**Keywords:** randomized clinical trial, RCT, Class V, bonding, adhesion, clinical effectiveness, NCCL, non-carious cervical lesions, composite restoration

## Abstract

**Purpose:**

This randomized controlled trial aimed to evaluate the 14-year clinical performance of a HEMA-free 1-step self-etch adhesive (1SEa) compared with that of a 3-step etch-and-rinse adhesive (3E&Ra).

**Materials and Methods:**

267 non-carious cervical lesions in 52 patients were restored with the microhybrid composite Gradia Direct (GC), bonded in random order either with the HEMA-free 1SEa G-Bond (GB; GC) or the 3E&Ra Optibond FL (OFL; Kerr), which is considered the gold-standard E&Ra (control). The restorations were followed over 14 years for retention, marginal adaptation and discoloration, and caries occurrence. Statistical analysis involved a logistic regression model with generalized estimating equations (2-way GEE model).

**Results:**

The patient recall rate at 14 years was 63%. In total, 79 restorations (39 GB, 40 OFL) failed because of retention loss (GB: 19.4%, OFL: 19.6%), severe marginal defects, discoloration and/or caries (GB: 21.7%; OFL: 22.5%). The overall clinical success rate was 58.9% and 57.9% for GB and OFL, respectively. The number of restorations with an unacceptable marginal defect (GB: 14.5%; OFL: 19.2%) and deep marginal discoloration (GB: 18.2%; OFL: 13.2%) increased during the last 5 years. No significant difference in overall clinical performance was recorded between the two adhesives (p > 0.05). Changes in the medical health of some patients and recurrence of abrasion/erosion/abfraction increased the failure rate and retention rate.

**Conclusion:**

After 14 years, restorations bonded with the HEMA-free 1SEa performed as well as those bonded with the 3E&Ra gold standard. Unacceptable marginal deterioration was the main reason for failure, followed by loss of retention.

About two decades ago, one-step self-etch adhesives (1SEas) were introduced on the dental market because of the faster application procedure and ease of use. This is possible, as 1SEas combine the etching, priming, and bonding functions in one application step, without a water-rinsing phase.^42^ The disadvantages of 1SEas are that they contain proportionally less resin and more solvent than when the primer is separate from the adhesive resin. Their film thickness is commonly below 10 µm, leading to suboptimal polymerization based on polymerization inhibition by oxygen, suboptimal stabilization of the adhesive interface, and reduction of the adhesive layer’s ability to absorb stress imposed by the shrinking restorative composite overlying it.^35,41,43^ 1SEas are more hydrophilic, absorb more water, and are less hydrolytically resistant. Currently, 1SEas are being replaced by “universal adhesives”, which are available in different application modes: self-etch, etch-and-rinse, and self-etch with prior selective etching of the enamel with phosphoric acid.^42^

G-Bond (GB) (GC; Tokyo, Japan), launched in 2004, is one of the first 1-step self-etch adhesives and is still available on the dental market. It is a mild SEa that creates an adhesive layer with a thickness of 10–15 µm. The pH of the primer is 2. The adhesive is HEMA-free, which requires strong air blowing after application to avoid the formation of water droplets at the interface due to phase separation.^35,38,39^ The in-vitro bond durability of GB to dentin is inferior to that of the gold-standard adhesives, 3E&Ra Optibond FL (OFL, Kerr; Orange, CA, USA) and the mild 2SEa Clearfil SE Bond (CSE, Kuraray Noritake; Tokyo, Japan).^3,5, 15,25,34^ However, several short- to medium-term clinical studies (3–9 years) evaluating the bonding efficacy of GB in NCCLs revealed favorable results.^2,19,24,29^ In 1996, seventeen years ago, a randomized clinical trial was started to evaluate the bonding effectiveness of GB in non-carious cervical lesions and to compare this with the gold standard 3E&Ra OFL. The 1-year, 3-year, 5-year, and 9-year data have been published.^24,34,36,37^ As the results of the 9-year recall were still favorable, it was worth doing a longer-term evaluation after 14 years of clinical functioning.^24^ To our knowledge, this is the longest-term follow-up of adhesives in NCCLs available in the literature. This study aimed to evaluate the 14-year clinical effectiveness of the HEMA-free 1SEa (G-Bond, GC) in NCCLs compared to that of the 3E&Ra (Optibond FL, Kerr). The null hypothesis tested was that the clinical performance of G-Bond would be inferior to that of OFL.

## Materials and Methods

The present study is a monocenter randomized clinical trial. At its inception 17 years ago, it was approved by the Ethics Committee of UZ Leuven (ML3306). Fifty-two patients were selected with 267 non-carious cervical lesions that needed a Class-V composite restoration. Written informed consent was obtained from each individual participant included in the study. All lesions that needed a composite restoration were included in each patient, starting from 2 lesions per patient. Half of the lesions were restored with the 1SEa GB and the other half with the 3E&Ra adhesive OFL (control adhesive). The adhesives GB and OFL were applied randomly using a randomization table. A total of 133 GB and 134 OFL restorations were placed in combination with the microhybrid composite Gradia Direct (GC). Detailed information regarding patient and lesion selection as well as randomization is described in the 1-year report.^37^

Two operators who were enrolled in a Master-after-Master in Restorative dentistry program placed the restorations. The detailed restorative protocol is described in the previous reports.^34,36,37^ Regarding tooth preparation, the dentin surface was roughened and an enamel bevel was prepared (1-2 mm). Most teeth (n = 213) were isolated with a plastic contour strip (contour strip, Ivoclar Vivadent; Schaan, Liechtenstein) and fixed with wooden wedges and cotton rolls. Alternatively, when it was impossible to place a contour strip (eg, no adjacent tooth), a retraction cord was used (n = 54). The adhesive was applied according to the manufacturer’s instructions (Table 1). Then, the composite Gradia Direct (GC) was placed in layers in a cervico-incisal direction and light cured. Finally, the restorations were finished and polished.

**Table 1:** Composition and application procedure of the adhesives tested

Adhesive	Manufacturer	Class	pH primer	Composition	Application procedure
G-Bond (GB)	GC; Tokyo, Japan	1SEa	2	4-MET, phA-m, DMA, ethanol, water, filler, photo-initiator and stabilizer	1. Shake bottle 2. Dispense the adhesive in a clean dispensing dish (immediately before application on the tooth, for each restoration, use new adhesive) 3. Apply on enamel and dentin 4. Leave undisturbed for 10 s 5. Air blow with maximum pressure 6. Light cure for 30 s.
Optibond FL (OFL)	Kerr; Orange, CA, USA	3E&Ra	2	Etchant: 37.5% H_3_PO_4_ OFL Prime: HEMA, GPDM, MMEP, water, ethanol, CQ, BHT OFL Adhesive: bis-GMA, HEMA, GDMA, CQ, ODMAB, filler (fumed SiO, barium, aluminoborosilicate, Na_2_SiF_6_), coupling factor A174	1. Apply etchant on enamel and dentin, let sit for 15 s, rinse for 15 s and dry for 5 s; 2. Apply primer while gently massaging 3. Dry for 5 s 4. Apply bonding in a uniform layer 5. Light cure for 30 s

BisGMA: bisphenol-A-diglycidyl methacrylate; BHT: butylhydroxytoluene, CQ: camphoroquinone; DMA: dimethacrylates; GDMA: glycerol dimethacrylate; GPDM: glycerol phosphate dimethacrylate; HEMA: 2-hydroxyethyl methacrylate; 4-MET: 4-methacryloxyethyltrimellitic acid; MMEP: mono-2- methacryloyloxyethyl phthalate; ODMAB: 2-(ethylhexyl)-4-(dimethylamino)benzoate; phA-m: phosphoric acid ester monomer.

### Evaluation Criteria and Procedure

**Fig 1:**
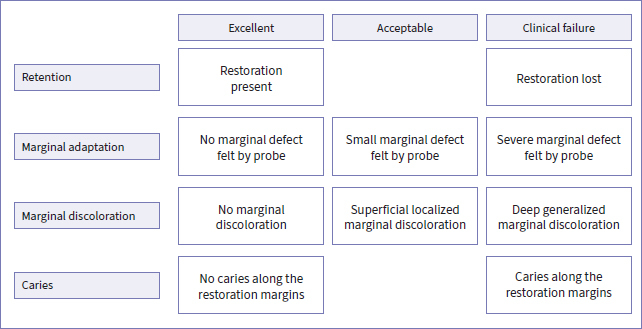
The four parameters determining the overall clinical success rate: retention, marginal adaptation, marginal discoloration, and occurrence of caries. Lost restorations, restorations with a severe marginal defect, deep marginal discoloration, and caries along the restoration margin were scored as clinical failure.

The restorations were evaluated by two independent dentist-examiners (MP, BVM) who were not the operators and were fully blinded to the adhesive used. Examination occurred at baseline and 6 months, 1, 2, 3, 5, 9, and 14 years of clinical service. The four key parameters evaluated were retention, marginal integrity, marginal discoloration, and caries occurrence and were used to determine the overall clinical success rate (Figure 1).^31^ Retention loss, severe marginal defects, deep marginal discoloration, and occurrence of caries were scored as clinical failure. Further details regarding the evaluation criteria are presented in the 1-year report.^37^

### Statistical Analysis

The clinical effectiveness of both adhesives was compared for the different key parameters as described in the evaluation criteria. A logistic regression model with generalized estimator equations (2-ways GEE model), using a compound symmetry structure for the working correlation matrix, was used to account for the clustered data (multiple lesions per patient).^31^ The analyses were performed using a statistical software package (Geepack library and R 2.13.2, R Foundation for Statistical Computing; Vienna, Austria). The odds ratio and 95% confidence intervals were determined. To observe the strength of the patient factors, a statistical sensitivity analysis was performed for the retention, failure, and perfect margins results. One lesion treated which each adhesive was randomly selected for each patient, and a McNemar test at a significance level of 5% (p < 0.05) was performed. This was carried out 100 times.

The effect on the retention and failure rate of having multiple restorations per patient vs only 2 restorations per patient was compared. In each patient, a restoration of each group was selected (GB and OFL) at random, and the failure rate and retention rate were calculated. This was done for 100 combinations, of which the mean failure and retention rates were calculated.

## Results

**Fig 2:**
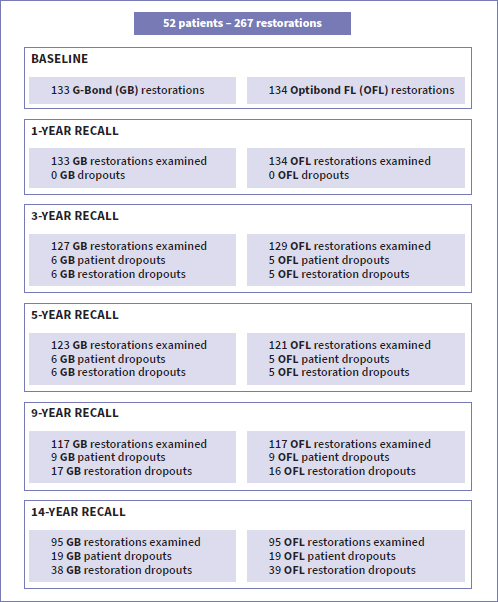
CONSORT flow diagram.

A CONSORT flow diagram with the number of patient- and restoration dropouts at each recall is shown in Fig 2. An overview of the results for the different evaluation parameters at each recall is presented in Table 2. The statistical analysis results (odds ratio and p-values) for the different evaluation parameters are detailed in Table 3. Table 4 shows the relationship between the degree of sclerosis, cervico-incisal height, maxilla/mandible, and the number of lost restorations at the 14-year recall (2-way GEE statistical analysis).

**Table 2:** Results in % at each evaluation period

	Baseline		1 year		3 years		5 years		9 years		14 years	
	GB	OFL	GB	OFL	GB	OFL	GB	OFL	GB	OFL	GB	OFL
Recall rate (restoration level)	100	100	100	100	94.7	94.0	89.5	90.3	87.9	87.3	71	71
Retention rate	100	100	98.5	99.3	94.4	96.0	91.6	93.4	89.7	89.7	80.6	80.4
Absence of marginal defect	97.7	98.5	42.0	60.9	22.7	62.8	20.2	44.2	2.6	13.7	1.3	4.1
Enamel marginal defect	0.0	0.0	51.9	26.3	70.6	22.3	72.5	34.5	95.3	65.4	96.1	90.4
Small enamel marginal defect	0.0	0.0	51.9	26.3	70.6	22.3	72.5	33.6	92.4	59.6	84.3	74
Severe enamel marginal defect	0.0	0.0	0.0	0.0	0.0	0.0	0.0	0.9	2.9	5.8	11.8	16.4
Dentin marginal defect	2.3	1.5	20.6	21.8	31.9	23.1	40.4	38.1	70.5	67.3	85.5	91.8
Small dentin marginal defect	2.3	1.5	20.6	21.8	31.9	23.1	40.4	37.2	66.7	66.3	77.6	85
Severe dentin marginal defect	0.0	0.0	0.0	0.0	0.0	0.0	0.0	0.9	3.8	1.0	7.9	6.8
Absence of marginal discoloration	100	100	88.6	94.0	64.7	86.8	54.1	76.1	28.2	43.6	13	27.6
Superficial localized marginal discoloration	0.0	0.0	11.5	6.0	35.3	13.2	42.2	23.0	59.6	44.2	68.8	59.2
Deep generalized marginal discoloration	0.0	0.0	0.0	0.0	0.0	0.0	4.6	1.8	8.7	6.7	18.2	13.2
Sensitivity	30.1	31.3	10.5	9.7	2.5	3.4	0.9	2.7	7.7	7.7	18.9	6.8
Absence of marginal caries	100	100	100	100	100	100	100	100	99.0	99.0	97.9	97.9
Overall clinical success rate	100	100	98.7	100	94.4	96.0	87.4	90.9	80.3	79.5	58.9	57.9

GB: G-Bond (1SEa); OFL: Optibond FL (3E&Ra).

**Table 3:** Comparison of the 14-year key parameters for clinical success of 1SEa GB versus 3E&Ra OFL (2-way GEE statistical analysis)

Parameter	OR	LL	UL	p-value
**Primary parameters**				**p-value+ Bonferroni correction**
Retention	1.0025	0.5192	1.9359	>0.999
Failure	1.0084	0.5364	1.8959	>0.9999
Caries	0.6607	02009	2.1737	>0.9999
Absence of marginal defect	1.0026	0.5192	1.9359	>0.999
Absence of marginal discoloration	1.5955	0.7668	3.3194	>0.999
**Secondary parameters**				**p-value**
Absence of dentin marginal defect	1.0025	0.5192	1.9359	0.9940
Absence of enamel marginal defect	1.0025	0.5192	1.9359	0.9940
Severe dentin marginal defect	1.0025	0.5192	1.9359	0.9940
Severe enamel marginal defect	1.0025	0.5192	1.9359	0.9940
Deep generalized marginal discoloration	0.7756	0.3573	1.6837	0.5205
Marginal discoloration on dentin side	1.3209	0.7877	2.2151	0.2914
Marginal discoloration on enamel side	0.3557	0.1696	0.7463	0.0063
Deep generalized marginal discoloration on dentin side	0.9442	0.4843	1.8408	0.8661
Deep generalized marginal discoloration on enamel side	0.4362	0.1407	1.3528	0.1508
Sensitivity	0.3041	0.1218	0.7592	0.0108

Regarding the parameters “marginal defects” and “marginal discoloration’”, only the results for retained restorations were compared. LL: Lower Limit of the 95% confidence interval; OR: Odds Ratio (ratio of the odds of the event for GB compared to the odds for OFL); UL: upper limit of the 95% confidence interval.

**Table 4:** Relationship between some secondary parameters of NCCLs and number of failures at the 14-year recall (2-way GEE statistical analysis)

	GB lost	OFL lost	Total no. of failures
Cervico-incisal height			
<2.5mm	4	8	67
≥2.5mm	14	10	82
Degree of sclerosis			
No sclerosis or slightly sclerotic	8	13	84
Intermediately sclerotic or strongly sclerotic	10	5	65
Location			
Maxilla	6	3	82
Mandible	12	15	67
**2-way GEE analysis**		**p-value**	
Cervico-incisal height of lesion		0.573	
Baseline group		0.994	
Cervico-incisal height : baseline group		0.074	
Sclerotic lesion		0.022*	
Baseline group		0.994	
Sclerotic lesion : baseline group		0.326	
Mandible		0.994	
Baseline group		0.0058**	
Mandible : baseline group		<0.114	

### Recall Rate

The overall restoration recall rate at 14 years was 71%. In total, 180 restorations were recalled (95 GB, 95 OFL). The overall patient recall rate was 63%; 43 out of 52 patients were examined. Four patients passed away, 4 patients were not able to come due to health problems, 4 patients were not reachable by phone or mail, 3 patients did not want to participate due to the COVID-19 crisis, and in 3 patients the restored teeth were extracted due to other reasons than restoration failure. Finally, in one patient, the fillings were covered with composite restorations that were placed to lengthen the teeth to increase the vertical dimension of occlusion.

In 6 patients who were seen at the 14-year recall, a dropout of 9 restorations was recorded (4 OFL, 5 GB) due to placement of a crown (4 teeth), extraction (1 tooth), and repair of composite restorations by another dentist (4 teeth). In 8 patients, who were not seen at the 14-year recall and whose restorations were recorded as failures at the previous recalls, these failed restored teeth (11 teeth; 1 with severe marginal defect, 2 teeth with severe microleakage, 2 teeth with severe marginal defect and severe microleakage, 7 lost restorations) were included in the 14-year recall.

### Clinical Success Rate

After 14 years of clinical service, this study recorded 79 failures: 39 GB restorations and 40 OFL restorations. The clinical success rate in both groups was similar: 58.9% for GB and 57.9% for OFL (p > 0.05) (Tables 2 and 3).

### Retention Rate

Thirty-six restorations (18 GB, 18 OFL) were lost at the 14-year recall, resulting in a retention rate of 80.6.% (GB) and 80.4% (OFL)(p > 0.05) (Tables 2 and 3).

### Marginal Integrity

One GB (1.3%) and 3 OFL (4.1%) restorations showed no marginal defects (p > 0.05). About 84.2% of GB restorations and 76.7% OFL restorations showed a clinically acceptable marginal defect. Regarding the restorations with an unacceptable marginal defect, 14.5% GB and 19.2% OFL restorations showed an unacceptable marginal defect on the enamel and/or dentin side (p > 0.05).

Regarding the location of the marginal defect, about 97% (GB: 98.7%; OFL: 96.1%) of the restorations showed a marginal defect (small and severe defects) on the enamel and/or dentin side. The percentage of GB and OFL restorations with a marginal defect (small and severe defects) on the enamel side was 96.1%/90.4% respectively, while 85.5% of the GB restorations and 91.8% of the OFL restorations showed a marginal defect (small and severe defects) on the dentin side (p > 0.05; Tables 3 and 4).

### Marginal Discoloration

No marginal discoloration was observed in 13% of the GB and 27.6% of the OFL restorations. The difference between both groups was not significant (p > 0.05). The percentage of restorations with deep marginal discoloration at the 14-year recall is 18.2% for GB and 13.2% for OFL (p > 0.05). Regarding the location of the marginal discoloration, GB shows significantly more leakage on the incisal enamel side (72.7% vs 46.1%; p = 0.006). On the dentin side, the percentage of marginal discoloration was about 32% for both groups (GB: 32.5%; OFL: 32.9%; p > 0.05).

### Caries and Sensitivity

In total, 4 restored teeth (2 GB 2 and OFL) showed caries at the restoration margin (p > 0.05). Three carious lesions (1 GB and 2 OFL) were observed in one patient. Two of these lesions had already been recorded at the 9-year recall.

An increase in tooth sensitivity since the 9-year recall was observed in the GB group (18.9%), while the frequency of increased tooth sensitivity remained the same in the OFL group (6.8%). The difference between the two adhesives was significant (p = 0.01; Tables 3 and 4).

### Placing Multiple Restorations per Patient

Looking at the effect of placing multiple restorations per patient, the McNemar analysis recorded no significant difference in retention rate and failure rate between the GB and OFL restorations within one patient (p > 0.05). It was not possible to calculate this for the parameter “perfect margin”, as the number of restorations showing a perfect margin at the 14-year recall was too low.

**Fig 3:**
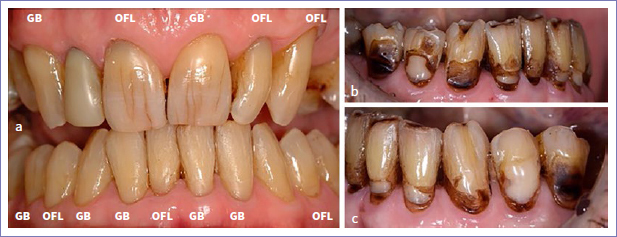
a. A 58-year-old male patient received 15 Class-V composite restorations at baseline. b and c. All restorations failed at the 14-year recall because the patient became severely medically compromised. The restorations on teeth 45, 43, 41, 33, and 35 were lost; severe clinically unacceptable marginal defects were noticed on teeth 44, 42, 31, 32, and 34.

**Fig 4:**
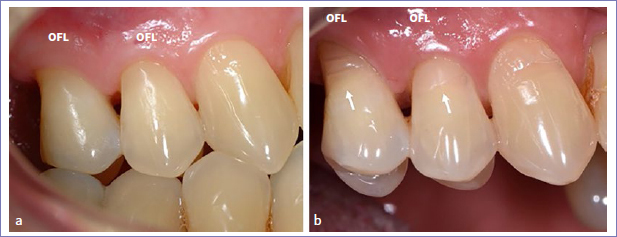
Baseline: the cervical lesions on teeth 24 and 25 were restored; b. 14-year control showing the presence of a severe marginal defect due to the recurrence of abrasion, abfraction, or erosion (white arrows). On tooth 25, the restoration was almost lost.

Regarding the placement of multiple restorations per patient, 5 patients received more than 10 restorations. Almost all restorations failed at the 14-year recall in two of these patients. In one patient, 11 out of 12 restorations failed; in the other, all restorations failed (15 out of 15; Fig 3). In a third patient, 4 out of 14 restorations were adjusted by his private dentist probably because of the presence of incisal wear. These restorations were considered as dropouts. In the same patient, 4 other restorations failed due to the recurrence of erosion, abrasion, and abfraction (Fig 4). In total, 12 restorations in 5 patients (6 GB and 6 OFL) were scored as clinically unacceptable due to recurrent abrasion, erosion, and abfraction resulting in unacceptable marginal defects.

**Fig 5:**
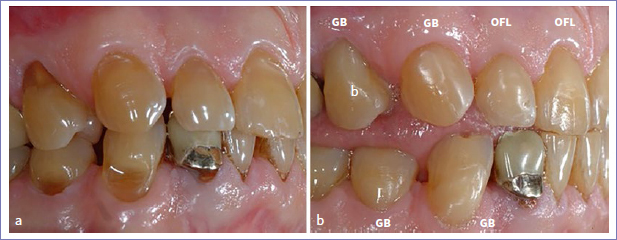
a. Baseline: a. A 65-year-old male patient had in total 11 cervical lesions that needed to be restored. b. 6-month recall of the cervical restorations on 12, 13, 14, 43, and 44.

**Fig 6:**
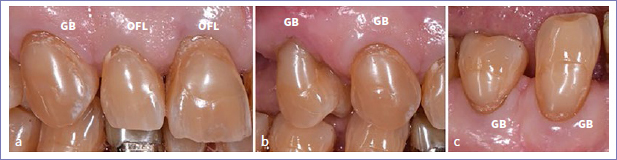
Restorations in Fig 5 at the 14-year recall. All 11 restorations in this patient were clinically acceptable. a: restorations on teeth 11, 12, 13; b: 13 and 14; c: 43 and 44. Wear of the composite and the presence of small but clinically acceptable marginal defects on the enamel and/or the dentin side are apparent. No difference in clinical performance was noticed between GB and OFL restorations.

**Fig 7:**
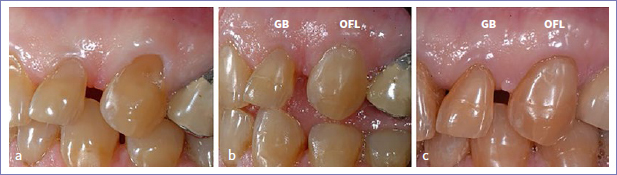
Same patient as in Figs 5 and 6. a. baseline; b. 6-month recall of the cervical restorations on teeth 22, 23; c. 14-year recall: both cervical composite restorations showed a clinically still-acceptable marginal defect.

Placing multiple restorations per patient in this study resulted in a lower retention and failure rate compared with placing two restorations per patient. The retention rate when two restorations were placed per patient is 86% for GB and 88% for OFL, with a variation between 75% and 95%, while the retention rate when multiple restorations per patient were placed was 80.6% for GB and 80.4% for OFL. Regarding the failure rate, this was 65% for GB and 71% for OFL (57%-84%) when placing two restorations per patient, while the failure rate when multiple restorations per patient were placed is 58.9% for GB and 57.9% for OFL.

### Secondary Parameters

The degree of sclerosis had an effect on the retention rate, but this was different for each adhesive (p = 0.022; Table 4). Eight GB and 13 OFL restorations failed when bonded to normal dentin and slightly sclerotic dentin, while 10 GB and 5 OFL restorations bonded to intermediately sclerotic/strongly sclerotic dentin were lost. The interaction effect is not significant (p = 0.326).

The operator did not have an effect on the retention rate (p = 0.94). For operator one, 44 out of 102 fillings failed (43%), which were equally distributed between GB and OFL (22/22). For operator two, 35 out of 88 fillings failed (40%), equally distributed between GB and OFL (18/17).

Regarding the restoration location (maxilla vs mandible), both adhesives showed significantly more failures in the mandible than the maxilla (p = 0.0058). The number of lost GB restorations is double in the mandible (12) compared to the maxilla (6). For OFL, the number of lost restorations in the mandible was 5 times higher than in the maxilla.

Regarding the size (cervico-incisal height) of the lesion, GB shows more lost restorations in large cavities (14 restorations >2.5 mm; 4 restorations <2.5 mm), while this effect was not observed for OFL (10 restorations >2.5 mm; 8 restorations <2.5 mm). The interaction effect was almost significant in a 2-way GEE model (0.074).

## Discussion

The present study evaluated the clinical performance of NCCL composite restorations, bonded with GB or OFL, after 14 years of clinical service. To our knowledge, this is the longest term follow-up of NCCL restorations published in the literature. The patient recall rate was 63%. A higher recall rate could not be reached, as some patients (n = 3) did not want to participate due to concerns over the COVID-19 crisis. In addition, 27 patients out of 52 were ≥74 years old at the 14-year recall. Four elderly patients passed away during the last 5 years, while 4 others became severely medically compromised and could not attend the recall. Therefore, it is recommended that when planning a long-term (>10 years) clinical trial of NCCL restorations patients, should be between 18 and 60 years of age.

In this study, the evaluation criteria of Vanherle et al^31^ were used instead of the FDI criteria.^13,14^ In 2006, when the study started, the FDI criteria were not yet available. The FDI criteria were first published in 2007, updated in 2010 and again in 2022.^12-14,18^ The FDI criteria, commonly used today in clinical trials, are more refined than those used in the present study.^17^ For each criterium, more subcategories can be distinguished. In addition, more evaluation criteria can be included depending on the aim of the study. The parameters evaluated in the present study, such as retention, marginal adaptation, caries, marginal discoloration, and postoperative sensitivity, were also evaluated in other in-house NCCL clinical trials initiated before 2007.^10,19,21,22^ For the different parameters, a distinction was made between excellent, clinically acceptable, and clinically not acceptable or failed. For the failed restorations, no further distinction was made between reparable or replacement needed. In the last update of FDI criteria, however, the importance of adding information on how to maintain the restorations – eg, by monitoring, refurbishing, repairing, and replacing – was emphasized.^12,18^

The parameter missing in the present study is the recurrence of erosion, abrasion, and abfraction. By further analysis of the restorations on clinical photographs, it was observed that 12 restorations (6 GB and 6 OFL) in 5 patients failed for this reason. The recurrence of erosion, abrasion, and abfraction contributed to the presence of an unacceptable marginal defect or even partial loss of the restoration (Fig 4). These restorations were scored as failed, although loss in bonding efficacy was not the main reason for the failure of these restorations.

The retention rate (GB: 80.6%; OFL: 80.4%) and success rate (GB: 58.9%; OFL: 57.9%) for both adhesives decreased further during the last 5 years. Unacceptable marginal deterioration, such as the presence of a severe marginal defect (GB: 14.5%; OFL: 19.2%) and/or deep marginal discoloration (GB: 18.2%; OFL: 13.2%), was the main reason for failure. At the 14-year recall, about 84.2% GB restorations and 76.7% OFL restorations showed a clinically acceptable marginal defect (p > 0.05). No difference was noted between both adhesives regarding the location of a marginal defect (enamel side [GB: 96.1%; OFL: 90.4%]) vs dentin side [GB: 85.5%; OFL: 91.8%]; Figs 5-7). Very few restorations exhibited no marginal defect (GB: 1.3%; OFL: 4.1%), while a higher percentage of restorations showed no marginal discoloration (GB: 13%; OFL: 27.6%; p > 0.05). Regarding the location of marginal discoloration, a significantly higher percentage of GB restorations showed marginal discoloration on the enamel side compared to the OFL restorations (GB: 72.7%; OFL: 46.1%) (p = 0.0063). A similar observation was made during the previous recalls. This was explained by the weaker etching effect of GB (pH=2), resulting in lower bond strength to enamel compared to that obtained by etching enamel with 35% phosphoric acid.^35^ This shortcoming is well known for mild and ultra-mild self-etch adhesives and can be solved by selective etching enamel with 35% phosphoric acid prior to application of the self-etching primer.^10,19,20,22,28^

Comparing the 14-year retention and success rate of GB and OFL with other long-term clinical trials, only a few long-term NCCL clinical trials are available for OFL. Three out-of-house studies mentioned retention rates for OFL after 12 to 13 years varying from 40%^30^ to 89%^44^ to 97%.^1^ In two of these studies, the predecessor, Optibond dual cure (Kerr), was used.^30,44^ The failure rate was not mentioned and the study design did not follow the guidelines that are currently required when starting a prospective randomized clinical trial.^12-14,18,26^ It is essential to follow these standardized guidelines to facilitate the publication of a profound systematic review and meta-analysis on the bonding efficacy of adhesives in NCCL clinical trials. Luhrs et al^16^ evaluated OFL in NCCLs after 7 years, with a retention rate of 82.8%. Only one additional restoration failed due to severe marginal discoloration. The adhesive in that study was tested in different cavity preparation designs.^16^

The present study found higher scores when comparing the failure rate and retention loss of GB and OFL with the results of in-house long-term clinical trials evaluating different adhesives, including OFL (Table 5). Several explanations can be given for this observation.

**Table 5:** Overview of long-term in-house clinical trials evaluating the bonding efficiency of adhesives in NCCLs

Adhesive	Follow-up period	Number of restorations per patient	Retention	Success (AFR)	Absence marginal defect	Absence marginal discoloration	Caries	Deep generalized marginal discoloration	Severe dentin marginal defect	Severe enamel marginal defect
OFL^21^	12 years	2 restorations per patient	94%	88% (1%)	12%	45%	0%	6%	0%	3%
PMQ-M^21^	12 years	2 rest per patient	90%	78% (1.8%)	12%	29%	0%	6%	0%	6%
PMQ-H^21^	12 years	2 rest per patient	85%	74% (2.2%)	9%	50%	0%	7%	3%	8%
CSE-E^22^	13 years	Max. 2 restorations per group per patient	96%	93% (0.5%)	16%	47%	100%	0%	0%	4%
CSE-NE^22^	13 years	Max. 2 restorations per group per patient	96%	86% (1.1%)	4%	41%	4%	4%	11%	0%
OFL^23^	6 years	Multiple restorations per patient	88.9%	80.9% (3.2%)	10.2%	66.7%	4.1%	3.1%	2.7%	2.7%
OXTR^23^	6 years	Multiple restorations per patient	92.9%	81.9% (3%)	15%	62%	5.4%	0.8%	7.9%	0%
OFL*	14 years	Multiple restorations per patient	80.4%	57.9% (3%)	4.1%	27.6%	1%	13.2%	6.8%	16.4%
GB*	14 years	Multiple restorations per patient	80.6%	58.9% (2.9%)	1.3%	13%	1%	18.2%	7.9%	11.8%

AFR: Annual Failure Rate; OFL: Optibond FL (Kerr); PMQ-M: Permaquick + Amelogen Microfill (Ultradent); PMQ-H: Permaquick + Amelogen Hybrid (Ultradent); CSE-E: Clearfil SE Bond with prior selective enamel etching with phosphoric acid (35%; Kuraray Noritake); CSE-NE: Clearfil SE Bond (Kuraray Noritake); OXTR: Optibond XTR (Kerr); GB: G-Bond (GC); *present study.

The clinical experience of the operator might have influenced the retention and failure rates. In the present study, the restorations were placed by dentists with 2 to 3 years of clinical experience. There was no difference between the two operators regarding retention loss of the restorations (p > 0.05). In the other in-house long-term clinical trials evaluating OFL and CSE, the restorations were placed by clinicians with a longer clinical experience (>10 years).^21,22^ In a 3-year clinical trial, Scotti et al^27^ observed that the operator’s experience had a significant influence on the retention rate of NCCL restorations bonded with OFL. The more experienced clinician obtained a higher retention and success rate compared to the less experienced clinicians. Similarly, a recently published systematic review evaluating the longevity of composite restorations recorded that the experience of the operator significantly determines the clinical performance of the adhesive restorations with time.^7^

Multiple restorations were placed per patient in the present study. This had a negative influence on the retention (GB=80.6%, OFL=80.4%) and failure rate (GB=58.0%, OFL=57.9%). The retention rate in patients when two restorations were placed was 86% for GB and 88% for OFL (75-95%). The failure rate was 65% for GB and 71% for OFL (57-84%). A 6-year in-house clinical trial comparing Optibond XTR (Kerr; Orange, CA, USA) and OFL in NCCL lesions followed a similar study design.^23^ The success and retention rate of OFL in this study were also lower than the results of the first-published 13-year clinical trial, where only 2 or 4 restorations were placed per patient. In this later study, a retention and success rate of 94% and 88% respectively was obtained for OFL (Table 5).^21^

In the present study, almost all restorations failed in two patients who had more than 10 restorations each. Both patients became medically compromised during the last 5 years. Fifteen restorations were placed in one male patient, but due to chemotherapy and radiotherapy, the quality of his restorations was severely impacted. All restorations became clinically unacceptable (Fig 3). Another female patient with 12 restorations became less mobile with aging; she had xerostomia, and was not able to brush her teeth well because of arthrosis. This resulted in the failure of 11 restorations; 7 restorations were lost, and a caries lesion was present at the margin of 3 restorations. In a third patient, where 14 restorations were placed at baseline, 4 restorations were scored as dropouts, as the restorations were repaired by his private dentist (probably due to incisal wear). Another 4 restorations in this patient were recorded as unacceptable due to a severe marginal defect or partial loss of the restoration mainly caused by recurrence of abrasion, erosion and/or abfraction (Fig 4). A similar phenomenon was observed in 8 restorations in 4 other patients. From these observations, we can conclude that patients’ risks appear to affect a restoration’s longevity. The importance of the patient factor is also well described in the systematic review by Demarco et al,^7^ which evaluated the durability of direct composite restorations.

Finally, it must be mentioned that 11 restorations which failed during the previous recalls and were not seen at the 14-year recall were included as failed restorations. This could also have resulted in a lower success and retention rate.

Based on the results of in-vitro studies evaluating the bond durability of GB and OFL to dentin, the long-term clinical performance of the restorations bonded with the 3E&Ra gold standard OFL was expected to be superior to that of the GB restorations.^3,5,6,15,25,34,42,43^ In the present study, however, the clinical performance of the NCCL restorations bonded with GB and OFL was equal after 14 years of clinical functioning. G-Bond, classified as a mild self-etch adhesive with a pH ≈2, contains the functional monomers 4-MET (4-methacryloxyethyl trimelletic acid) and a phosphoric-acid ester monomer, most likely 10-MDP (10-methacryoloxydecyl dihydrogen phosphate), although the latter is neither confirmed nor denied by the manufacturer. 10-MDP is the most effective commercially available monomer, able to form stable monomer-Ca bonds and nanolayering, contributing to the durability of the bond.^11,45-47^ In addition, the HEMA-free GB requires strong air drying after application to remove water which separates from the more hydrophobic components from the interfacial area, enabling better polymerization. Such strong air drying is more effective on a relatively flat surface, as is present in a NCCL, than in a complex Class-I or -II cavity.^40^

The only differences between the two adhesives at the 14-year recall are that GB restorations exhibited significantly more marginal discoloration on the enamel side (p = 0.006), as mentioned above, and that the restored teeth showed significantly more hypersensitivity (p = 0.01). The increased hypersensitivity, which occurred during the last 5 years, is not related to the adhesive and can be explained by gingival retraction, recurrence of erosion, abrasion and/or abfraction observed on several restored teeth.

This study’s results, which show no significant difference in clinical performance between the HEMA-free 1-step SE adhesive GB and the 3-step ER adhesive OFL, are in accordance with the conclusion of some recently published systematic reviews evaluating the bonding efficacy of adhesives in NCCLs. According to the systematic review by Dreweck et al,^9^ which compared the retention rates of 3-step ER and 1-step SE adhesives, there is no evidence that 3-step ER adhesives have better retention rates than 1-step SE adhesives.^9^ However, marginal defects and marginal discoloration were more often recorded for the 1SEa at 12–24 months. Another systematic review by da Silva et al^4^ reported similar clinical performance for HEMA-free and HEMA-containing adhesives in NCCLs. Finally, in a third systematic review, also by Dreweck et al,^8^ no evidence was available from randomized controlled clinical trials to support the widespread concept that the so-called gold standard adhesives, OFL and CSE, are better than other competitive brands available on the dental market. A significant difference was only found for OFL and the other adhesives at 60 to 96 months; however, only three studies were included in that meta-analysis.^8^ This underscores the need for long-term clinical trials (>10 years) evaluating the bonding effectiveness of adhesives in NCCLs.

Regarding the influence of the secondary parameters, the same observations were made at the 5- and 9-year recall: (1) significantly more lost restorations were observed for both adhesives in the mandible than in the maxilla (p = 0.0058), and (2) OFL works significantly better on sclerotic dentin than does GB (p = 0.022). The explanation for these phenomena has been described in detail in the 5- and 9-year follow-up.^24,34^

## Conclusions

After 14 years, restorations bonded with the HEMA-free 1SEa performed clinically equally to those bonded with the 3E&Ra gold standard. Unacceptable marginal deterioration was the main reason for failure, followed by loss of retention. Changes in medical health of some patients and recurrence of abrasion, erosion, and abfraction increased the failure and retention rate in this study.

## Clinical relevance:

The simplified mild HEMA-free 1SEa and the considered gold-standard 3E&Ra are clinically equally successful in NCCL composite restorations after long-term clinical functioning. The patient’s risks appear to be a factor affecting the long-term clinical performance of the NCCL restorations.
